# A Tool to Measure Young Adults’ Food Intake: Design and Development of an Australian Database of Foods for the Eat and Track Smartphone App

**DOI:** 10.2196/12136

**Published:** 2018-11-07

**Authors:** Lyndal Wellard-Cole, Melisa Potter, Jisu (Joseph) Jung, Juliana Chen, Judy Kay, Margaret Allman-Farinelli

**Affiliations:** 1 School of Life and Environmental Science The University of Sydney Sydney Australia; 2 School of Information Technology The University of Sydney Sydney Australia

**Keywords:** diet surveys, smartphone, mobile phone, young adult

## Abstract

**Background:**

Dietary assessment is reliant on the collection of accurate food and beverage consumption data. Technology has been harnessed to standardize recording and provide automatic nutritional analysis to reduce cost and researcher burden.

**Objective:**

To better assess the diet of young adults, especially relating to the contribution of foods prepared outside the home, a database was needed to support a mobile phone data collection app. The app also required usability testing to assure ease of entry of foods and beverages. This paper describes the development of the Eat and Track app (EaT app) and the database underpinning it.

**Methods:**

The Australian Food and Nutrient Database 2011-13, consisting of 5740 food items was modified. Four steps were undertaken: (1) foods not consumed by young adults were removed, (2) nutritionally similar foods were merged, (3) foods available from the 30 largest ready-to-eat food chains in Australia were added, and (4) long generic food names were shortened and simplified. This database was used to underpin the EaT app. Qualitative, iterative usability testing of the EaT app was conducted in three phases using the “Think Aloud” method. Responses were sorted and coded using content analysis. The System Usability Scale (SUS) was administered to measure the EaT app’s perceived usability.

**Results:**

In total, 1694 (29.51%) foods were removed from the Australian Food and Nutrient Database, including 608 (35.89%) ingredients, 81 (4.78%) foods already captured in the fast food chain information, 52 (3.07%) indigenous foods, 25 (1.48%) nutrients/dietary supplements, and 16 (0.94%) child-specific foods. The remaining 912 (53.84%) foods removed were not consumed by young adults in previous surveys or were “not defined” in the Australian Food and Nutrient Database. Another 220 (3.83%) nutritionally similar foods were combined. The final database consisted of 6274 foods. Fifteen participants completed usability testing. Issues identified by participants fell under six themes: keywords for searching, history list of entered foods, amounts and units, the keypad, food names, and search function. Suggestions for improvement were collected, incorporated, and tested in each iteration of the app. The SUS of the final version of the EaT app was rated 69.

**Conclusions:**

A food and beverage database has been developed to underpin the EaT app, enabling data collection on the eating-out habits of 18- to 30-year-old Australians. The development process has resulted in a database with commonly used food names, extensive coverage of foods from ready-to-eat chains, and commonly eaten portion sizes. Feedback from app usability testing led to enhanced keyword searching and the addition of functions to enhance usability such as adding brief instructional screens. There is potential for the features of the EaT app to facilitate the collection of more accurate dietary intake data. The database and the app will be valuable dietary assessment resources for researchers.

## Introduction

Obesity is a global problem and although overweight and obesity is prevalent across all adult age groups in Australia, younger adults are a very vulnerable group. For example, Australian adults aged 18-30 years have had the greatest increase in Body Mass Index per year of any adult age group [1].

Young Australians are also the group who spend the highest proportion of their household income on fast foods and eating outside the home [2]. Frequent consumption of fast foods has been linked to poorer quality diets and weight gain [3,4]. However, there is little national data on the contribution these foods make to overall diets. While countries such as the United States have detailed data on the location of the purchase and consumption of foods, Australia does not. Therefore, there is no available consumption data for foods purchased from cafes, bars, restaurants, fast food outlets, takeout shops, and other food outlets. Research is needed to determine whether foods purchased and eaten outside the home are having an impact on young people’s nutritional intake.

Dietary assessment is reliant on accurate unbiased collection of food and beverage consumption data. Typically, a participant must recall or record their dietary intake and provide portion sizes, which is a tedious process. In addition, to reveal the source of the meal preparation, further details must be recorded. The researcher must ensure the validity of the recording and convert dietary intake into nutrients. In recent years, technology has been harnessed to standardize recording and provide automatic nutritional analysis to reduce cost and researcher burden [5]. An example of technologies used to assist in recording intake is barcode scanning [6]. However, while scanning barcodes is useful in identifying foods purchased in packages, it is not applicable to most food prepared outside the home by the catering industry.

Recent studies have demonstrated that mobile phone apps can be valid measures of dietary intake at the population level [7-10]. However, a number of essential challenges must be addressed in the design of such apps. The starting point for creating an app for monitoring dietary intake must be the underpinning database of foods and beverages and their nutritional composition [11]. The usability of the interface for recording of foods is critical and equally important is the search functionality to support quick and easy locating of foods in the database. A comprehensive database of foods can be assembled, but it needs naming conventions that young adults readily recognize to enable better recording of the foods consumed [12]. Participants report that they never know if the food option they select is the appropriate one and they are confused by the large list of options to scroll through on an app like MyFitnessPal [12].

To better assess the diets of young adults in relation to the dietary contribution of foods prepared outside the home, a database was needed to support the development of a mobile phone app for dietary data collection. Assessment of potential participants’ ability to search for food items was also required. Recording of the location of food purchase and tagging of food items would provide further insight into the outlets associated with food purchase and/or consumption.

This paper describes the development of the Eat and Track mobile phone app (EaT app), including the database underpinning it, and provides insights for other researchers seeking to develop apps for recording dietary intake.

## Methods

### Context

The development of the EaT app and the database that underpins it is part of the Measuring Young adults’ Meals (MYMeals) Study, which aims to (1) determine how frequently young adults purchase and consume foods outside the home, and the types of foods they are purchasing and consuming, (2) the relative contributions of different food outlets to overall food and beverage intake, and (3) the extent that food and beverages consumed outside the home contribute to young adults’ total energy and nutrient intakes [13]. The EaT app will collect 3 consecutive days of dietary intake data and information on where foods and drinks were obtained. This will allow analysis of the impact of foods eaten outside the home (for full methods, see the MYMeals Study protocol [13]).

The AUStralian Food and NUTrient Database (AUSNUT), 2011-13 [14], was used as a basis for the EaT app. The AUSNUT database was developed to analyze the foods and beverages that were consumed during the 2011-12 National Nutrition and Physical Activity Survey (NNPAS) and National Aboriginal and Torres Strait Islander Nutrition and Physical Activity Survey [15]. The food item names are mostly unbranded, and there was not always a distinction in the names between homemade foods and foods prepared ready-to-eat. Many meals and snacks from ready-to-eat food outlets cannot be readily identified using this database.

The EaT app database development (Stage 1) and formative usability testing of the EaT app (Stage 2) were conducted sequentially, but changes to the database were incorporated as a result of the usability testing. However, for clarity the Methods and Results for each part have been presented together.

### Stage 1 Methods

#### Development of the Eat and Track App Database

The AUSNUT 2011-13 database [14] was used as the starting point of the EaT app database. The AUSNUT database contains the nutrition composition of 5740 foods. To modify the database, four steps were undertaken: (1) foods that would not be consumed by young adults were removed, (2) foods that were from the same subcategory food group and nutritionally very similar (±10% for energy and all 25 nutrients) and varieties of the same food were merged into one food item (eg, yellow and green apples), (3) foods available from the 30 largest ready-to-eat food chains in Australia were added, and (4) the long string generic names were shortened and simplified to terms in common usage in the Australian community.

The database was reviewed to remove foods that were not likely to be eaten by potential participants. These include foods available only in certain states outside the EaT app’s intended use (such as Indigenous foods available only in remote communities in the Northern Territory of Australia) [16], and food for specific groups outside of those who would be using the EaT app (eg, baby food and formula). The amounts of every food consumed by 18- to 30-year-olds during the NNPAS [17] were examined, and those that were not consumed were excluded. Examples of excluded foods included some offal meats and dietary supplements such as high-energy and high-protein formulas.

The AUSNUT food database organizes foods into three levels to assign each a unique ID: the major food group (eg, “Fruit products and dishes”), sub-major food group (eg, “Pome fruit”), and a minor category (eg, “apples”) [18]. From that, a name is assigned to each food within the category. To identify nutritionally similar foods, the database was analyzed at the minor level. Any foods that were within ±10% for energy per 100 grams and all 25 different nutrients per 100 grams were identified and combined.

Due to the long character-length of many names in AUSNUT database, food names were shortened and simplified by an Accredited Practising Dietitian according to how foods are commonly referred to in Australia. For example, “Soft drink, lemonade, regular” was simplified to “Lemonade.” Additionally, brand names were sourced from the AUSNUT 2011-13 Food Details File [19]. Some foods were renamed using multiple synonyms to increase likelihood of the end-user being able to find foods. For example, “Pizza, ham & pineapple” was replaced with “Ham & pineapple/Hawaiian pizza.”

In addition to the generic foods from the AUSNUT database, an additional 2229 foods obtained from the 30 largest ready-to-eat food chains in Australia were included. Chains included in the database included traditional fast food (eg, burgers, fried chicken, pizza), fast casual chains, ice cream shops, bakery and salad chains, and beverage only or café chains.

Nutrition information for the ready-to-eat food chains was obtained from the companies, using methods detailed elsewhere [20]. However, this fast food nutrition information encompassed only the nutrients that are mandatory for Nutrition Information Panels in Australia (ie, energy, protein, total and saturated fats, carbohydrates, sugars, and sodium) [21] and not the comprehensive information from the AUSNUT database. Information was downloaded from chain websites, obtained in store or by request from company customer service enquiries. Data were collected in 2015 and checked for availability before being included in the EaT database. This was conducted by reviewing the menus of all the included chains and removing any foods that were no longer available. This was undertaken by one author (LWC) who was an Accredited Practising Dietitian in January 2017. All foods were named to include the chain name in the food name for the app, for example “Big Mac burger, McDonald’s.”

#### Portion Sizes and Measurements

The EaT app database contains information on portion sizes and measures for all foods. All foods were assigned either grams or millilitres as the unit of measurement. This was used in the EaT app for entry of the amount of the food. In addition, portion sizes were sourced from the companion AUSNUT food measures database [22]. This contains commonly eaten portion sizes reported in the NNPAS, often in metric household measures (eg, cup, tablespoon) [22]. To make recording of foods easier, we calculated and added portion sizes for each food using commonly eaten portions, such as standard glass sizes for alcoholic beverages. Where necessary, the portion sizes were calculated from the standard food weights and/or densities from the AUSNUT 2011-13 food measures database [22].

#### Quality Checking

Initial data quality checks were conducted on the additional foods added to AUSNUT by 2 independent researchers. Suspected errors in data were cross-checked from the original nutrition information and followed up with the chain, if necessary. Any fast foods with incomplete nutrient information were omitted from the database. Portion sizes for all foods were reviewed by an additional researcher independent of the database development process to ensure errors were detected and rectified. A final review of the entire database was conducted by 2 researchers before it was provided to the app development team for integration into the app.

### Stage 1 Results

A total of 1694 foods (29.51%) were removed from the original AUSNUT database. Of the 1694 foods, 608 were ingredients that cannot be eaten without being made up (eg, dry soup mix or dried legumes and pulses) (35.89%), 81 were fast foods (4.78%) that were already captured in the fast food chain information, 52 were indigenous foods (3.07%), 25 were nutrients or dietary supplements (1.48%), and 16 were children’s foods (including human breastmilk, infant and toddler formulas, and baby foods) (0.94%). The remaining 912 foods (53.84%) either were not consumed by young adults in the NNPAS or were included in AUSNUT as “not defined” or “not specified.”

Once identified, foods that were within ±10% for energy and all 25 different nutrients were collapsed into single entries. For example, “apple, green” and “apple, golden” were combined into a single entry, “apple, green/golden.” When there were small differences in nutrient composition, the collapsed items’ nutrient contents were averaged. This process resulted in 220 foods (4%) being combined with other entries as they were nutritionally similar. The final database included in the EaT app consisted of 6274 foods, including 4046 foods (64%) from the AUSNUT database and 2229 branded ready-to-eat food chain items (36%).

### Stage 2 Methods

#### The Eat and Track App

The EaT app was designed to draw on lessons from the previously validated electronic Dietary Intake Assessment (eDIA) app [7,8]. The app was developed using React-Native, a cross-platform app development platform that generates apps for Android and iOS, which covers most mobile phone users. The EaT app stores every detailed interaction and food intake to a secure remote server in real time so that researchers are able to check and follow up logging. It was designed to reset the logging status every day at 3 a.m. to prevent participants from changing their data retrospectively.

**Figure 1:**
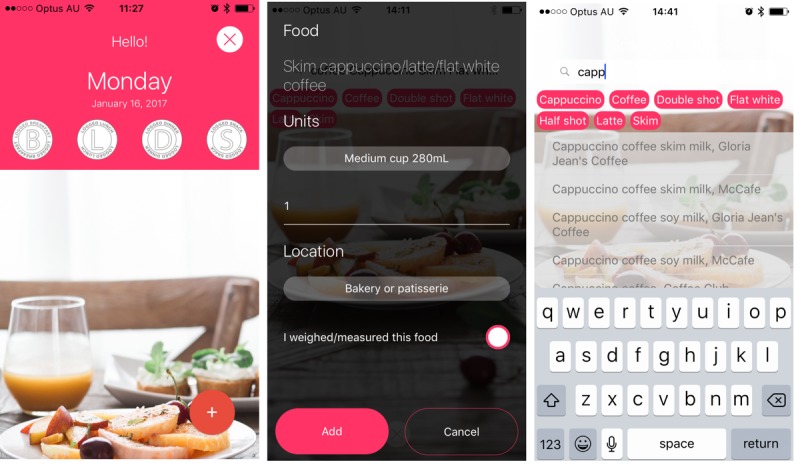
Eat and Track app screenshots.

The app interface (Figure 1) allows searches for common and brand names to increase potential for individuals to select appropriately matched foods. The interface was developed to include keyword functionality to improve food searches [13]. For example, when a user types “milk,” a shortlist of all milk types appears. Keywords were added to the database for the most commonly eaten foods identified from the Australian Health Survey [17]. After selecting a food or drink, participants then record the amounts consumed and the location where the food was sourced. If participants cannot find a food listed in the app, they can manually enter it as a new food.

#### Iterative Usability Testing of the Eat and Track App

Qualitative, iterative usability testing was conducted in three phases during EaT app development. The “Think Aloud” usability testing method [23] was used to gain insights from participants on usability issues, such as the ease of finding specific foods using the search function and selecting the correct portion size.

Participants were recruited face-to-face and from posters on the University of Sydney campus, social media posts, and advertisements on the university’s volunteer for a research study webpage. Potential participants were eligible if they were aged 18-30 years and could speak, write, and understand English. Participants were excluded if they had undertaken or were undertaking formal education in nutrition or information technology. Each participant was eligible to participate in only one phase of the study. Five participants were included in each phase and as an incentive they were entered in a prize draw for an Aus $50 gift voucher on completion of the study. Research has suggested that 80% of high severity usability issues can be determined with 5 participants, and 90% of issues with 10 participants [23-25]. Additionally, small samples are appropriate if there are to be multiple rounds of testing [24,25]. Therefore, 15 participants, 5 in each phase, were considered adequate for this study.

All participants provided consent. The usability studies were approved by the University of Sydney Human Ethics Research Committee (project number 2016/546).

**Figure 2:**
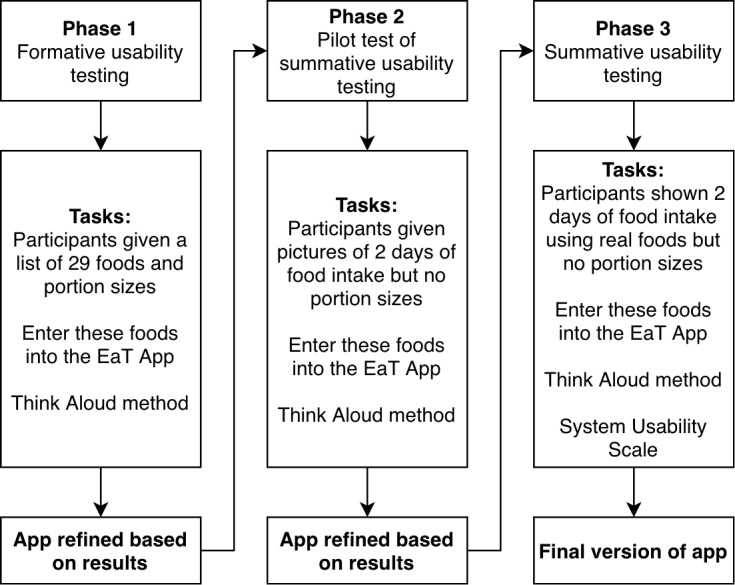
Iterative usability testing approach. EaT App: Eat and Track App.

In all phases, participants were provided with a device with the EaT app already installed to complete the testing. Participants were given information about the study and verbal instructions on how to use the EaT app and viewed a video demonstrating the Think Aloud method. All participants completed a demographic questionnaire. Figure 2 shows the three phases of the development.

In Phase 1, we tested as many of the features and functionalities of the app as possible, including how easily participants could find commonly consumed foods and enter correct portion sizes. Participants were provided with a list of 29 of the most commonly eaten foods for this age group from the latest Australian Health Survey [17] and relevant portion sizes and asked to search for the foods and enter these into the EaT app. Participants were instructed to verbalize their thoughts as they performed each task, but no assistance was provided. One researcher observed the participant, and another recorded the participants’ comments. Comments were also audio-recorded for future analysis. All questions raised by the participants were answered after the testing was complete. Modifications to the app were made based on feedback from this phase, before further testing in Phase 2.

Phase 2 was designed to test the search functionality and keywords. Five different participants completed Phase 2. Participants were again given a mobile phone with the revised EaT app pre-installed and photographs of a hypothetical 2 days of food intake, including breakfast, a morning snack, lunch, an afternoon snack, dinner, and an evening snack, including drinks. Participants were instructed to enter these foods into the EaT app and verbalize their thought processes and questions. Further modifications were made based on this phase for summative testing in Phase 3.

**Figure 3:**
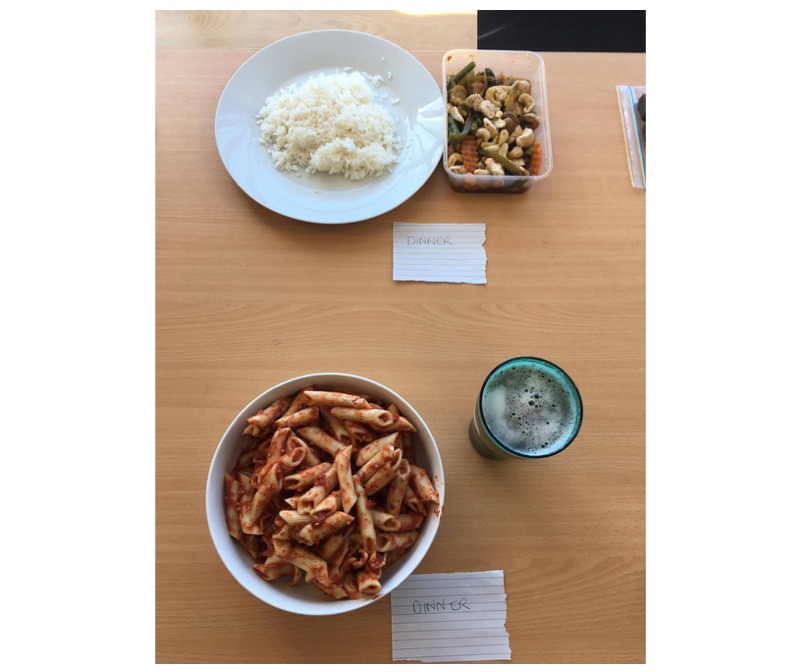
An example of the real foods presented to participants in Phase 3.

Phase 3 tested participants’ ability to estimate and enter portion sizes in a real-life situation and to test how long it would take to complete a day’s worth of logging using the EaT app. We also tested the app’s overall usability. In Phase 3, we presented 5 participants with 2 days of food intake using real foods that had been pre-weighed and measured. Each day consisted of breakfast, a morning snack, lunch, an afternoon snack, dinner, and an evening snack, including drinks (Figure 3). Participants were instructed to enter each food and drink into the EaT app as well as to estimate and enter the portion size for each food. The Australian Health Survey Food Model Booklet [26] containing to-scale images of different-sized foods and drinks was provided to help participants estimate portion sizes. The time it took participants to complete each task was recorded, to provide additional evidence about how difficult participants found the tasks.

An online version of the System Usability Scale (SUS) [27] was administered to participants in the third phase. The widely used SUS questionnaire measures a system/app’s perceived usability through a series of 10 five-point Likert scale questions [27]. Additionally, participants were asked to rate the ease of estimating portion sizes of foods and beverages on a seven-point scale (extremely difficult to extremely easy). To collect feedback for further improvements to the EaT app, participants were asked two open-ended questions on their overall likes and dislikes about the app.

#### Data Analysis

Audio recordings of the usability testing and the researchers’ observations were transcribed verbatim. Content analysis principles were used to sort and code data [28]. The transcription and observational data were reduced into themes that were identified a priori by the 2 researchers present in the usability testing sessions, in discussion with a third researcher. Once all the themes were identified, the data were aggregated to identify commonalities. Written data were then coded by a third researcher to ensure it was coded consistently.

The length of time taken for participants to complete each task, means, and standard deviations (SD) were calculated for each participant. Participants’ scores for each item in the SUS were calculated to provide a score from 0 (very difficult to use) to 100 (very easy to use) [29]. A score above 68 is considered average [30]. Qualitative findings from the open-ended questions, including the question on portion size were organized into themes.

### Stage 2 Results

A total of 15 participants aged 23-30 years completed the usability testing across the three phases. Eight participants were male. Nine participants had prior experience tracking health, nutrition, or physical activity behaviors.

#### Phases 1 and 2

Participants completed the tasks with varying levels of success (see Table 1). Common problems encountered by participants included incorrect spelling and/or punctuation despite being given the list of names with correct spelling (eg, omitting apostrophes or hyphens), foods being known as other names (eg, participants entering “hot chips” instead of “potato chips,” or “Coke” instead of “Coca-Cola”), and words used in descriptions of many foods appearing in many searches (eg, searching for “milk” as opposed to “Cow’s milk” resulted in “milk chocolate,” “milk coffee,” “coconut milk,” and “milkshake,” among others). Clear themes of usability problems emerged by the end of each iteration of testing, providing the basis for iterative refinement of the interface.

**Table 1:** Issues raised in Phases 1 and 2 of usability testing.

Success of participant and notes		Food item
**All participants found these items easily and without help**		
	All participants found these items, though often the keywords participants entered were different. For 3 foods, they did not come up as the first item in the list.	Black/green/chai tea no milk, regular/decaf; instant coffee, white Light/mid-strength beer; red wine Lemonade; lemon, lime and bitters; sports drink, bottled Orange fruit drink 25% juice; apple fruit drink Milo powder; drinking chocolate powder Vegemite spread; peanut butter Soy sauce; BBQ sauce; mayonnaise Iceberg lettuce; mandarin; green cucumber; strawberry; watermelon, peeled Cooked broccoli; cooked peas; cooked carrot, corn & pea/bean mix from frozen Garden salad with cheese Cooked white rice Garlic/herb bread Sweet plain biscuit, eg, Nice, Malt-o-Milk, Marie, Milk Arrowroot, plain Tiny Teddies, Morning Tea Milk chocolate Jelly lolly
**All participants found these items with some searching difficulties**		
	Participants who entered only part of the search term (eg, “fried egg” instead of “fried chicken egg”) had to scroll through a long list to find the item. Often there were no keywords to narrow the search.	Apple, red; orange Orange juice; apple juice Tomato sauce; honey; ground pepper Coles regular margarine spread Bacon middle rasher/shortcut fat trimmed, baked, roasted, fried, grilled or BBQed in butter/margarine Processed ham and chicken luncheon meat Fried chicken egg in butter Wholemeal bread
	Participants could not find the item if they entered “Coke.” One participant needed help as they misspelled “Coca” (“cocoa”).	Coca-Cola
	Some participants stated they would not think to enter “raw.” Entering only the keyword meant that participants had to scroll through a long list.	Raw banana Raw onion Raw carrot Raw avocado Peeled Desiree/Coliban/red skin potato, raw Raw common/Roma tomato
**Some participants had difficulty finding these items**		
	One participant suggested that a keyword for “sugar” would make the search easier.	Raw sugar
	Some participants could not find this term as they omitted the apostrophe. Other participants tried searching with “milk” and had to scroll through a long list to find the item.	Skim cow’s milk
	Some participants entered “ham” and had to scroll through a long list to find the item.	Leg ham
	If “weetbix” was entered without the hyphen, participants could not find the item and needed help.	Sanitarium Weet-Bix Original
	If “ice cream” was entered, participants had to scroll through a long list as the list was not in alphabetical order. One participant entered “icecream,” which returned no items.	Ice cream, all flavors
**Many participants had difficulty finding these items without help**		
	Most participants had trouble with this task. There was confusion with entering the “amount” and “unit.”	Tap water
	Some participants were confused by the many different spreads in the app, but eventually found the item after assistance from a researcher. Some participants entered what they would enter in a real-life situation. This included “butter,” which required extensive scrolling, and “margarine,” which did not yield the item at all.	Regular fat dairy blend spread, eg, Western Star spreadable, Coles Spreadable Dairy Blend, Beautifully Butterfully Premium
	Most participants had difficulty with this task. Some participants tried entering “hot chips” and were confused when the search returned no items.	Deep fried potato chips, from restaurant/takeout

**Table 2:** Issues and improvements identified in Phase 3 of usability testing.

Function	Participant quote	Issue
Keywords	“There is inconsistency in that some products have keywords and some don’t, for example, it would be helpful to have keywords for chips, such as ‘potato,’ ‘crisps,’ and ‘plain’ to help narrow the search down”	Not all foods have keywords Some participants did not notice pink keyword buttons Other participants did not know what pink keyword buttons were for
History of previously added foods	“I didn’t realize what the history list was, it wasn’t obvious and the list of foods looked exactly the same as the list of foods provided when you are searching for a food”	Several participants did not notice or use the history list
Amounts and units	“I find this function very unintuitive, I entered 600 under amount and then chose 600 mL bottle under unit, which actually meant that I had 600 x 600 mL bottles, which is not accurate”	Several participants found the order of the Amount and Unit fields confusing
Keypad	“I have entered orange juice into the search bar and it has given me a list of options to choose from, but I can’t figure out how to get the keypad out of the way so I can see the rest of the list”	Several participants did not know how to minimize the keypad
Food names	“It would be helpful if the app recognized different synonyms of for food names, eg, chips/crisps, Coca Cola/Coke and chips/fries”	Descriptions of foods inconsistent and confusing, making it harder to find the right item App does not recognize some common synonyms of food names (eg, chips/fries) App does not recognize the word “and” and the symbol “&” as the same thing
Search function	“Some items don’t appear at the top of the list even when you’ve typed the exact phrase as the item on the list appears (eg, orange juice—the item is half way down the list)”	App does not return 2-word matches as it does for single word matches

Modifications from Phase 1 included updating keywords to assist with searching for foods and drinks, and improving the search function to return single word matches first followed by all foods with the word entered appearing in alphabetical order. The feedback from Phase 2 led to several further updates to the app including improving the search functionality to ignore hyphens. Additionally, further improvements to the usability of the interface were also incorporated, including the addition of a sign-out button, changes to the look of the app, fixing app bugs, and allowing users to minimize the keypad so the shortlist was visible on the entire screen.

#### Phase 3

The mean time taken to enter 2 days of dietary intake into the EaT app in Phase 3 was 22 minutes, 13 seconds (SD 4.0). The slowest completion time was 30 minutes, 10 seconds, and the fastest completion time was 16 minutes, 22 seconds. Participants spent the least amount of time completing the task when they entered a fast food beverage and the most time completing the task was entering a pasta dish. Single food items were entered more quickly than mixed dishes or composite foods.

The Think Aloud protocol provided participants with the opportunity to provide insights into the difficulties they had and the reasons for these difficulties when using the app. They were also invited to make suggestions for improvements as they completed the usability testing. This highlighted several issues and improvements that would make using the app simpler. These are shown in Table 2.

Issues in this phase broadly fell under six themes: keywords for searching, the history list of previously entered foods, fields for amounts and units, keypad, food names, and search function. Some participants did not notice or understand the keyword buttons or the list of previously entered foods. The participants were unclear that there were two fields for amounts of food—one for the number and a separate one for the unit of measurement. Some participants could not minimize the keypad. Some still struggled with the food names, and even when a food was entered, the exact match did not necessarily show as number one in the options.

The EaT app obtained a mean SUS score of 69 (range 45-90), rating it about average for usability. Ten of the 15 participants (67%) rated it as above average for usability. After the third phase, we concluded that remaining usability problems were minor and the app was ready for use.

#### Final Eat and Track App

The final EaT app incorporated the Phase 3 feedback. Specifically, more refinements were made to food names, for example adding the term “hot chips” to the name of “potato chips” to reflect what participants searched for in the usability testing, or adding alternative spellings of food names to enhance searchability. Additional keywords were added to aid narrowing lists of foods. The “unit” and “amount” fields order were swapped, to enable participants to enter the correct amounts, and pop-up instructional screens were introduced at sign-in to provide participants with brief information on how to use the app.

## Discussion

### Principal Considerations

A food and beverage database, including comprehensive ready-to-eat food chain data, has been developed to underpin the EaT app. This will support data collection on the eating-out habits of 18- to 30-year-olds in New South Wales, Australia’s most populous state. The database development process has resulted in a database with commonly used food names and extensive coverage of foods from ready-to-eat chains. The usability testing allowed search and food naming issues to be identified and resolved, and refinement of app to improve usability.

Carter et al designed a database with inclusion of an extensive number of commercial foods available in the United Kingdom to support their online dietary assessment platform, myfood24 [11]. Including brand names is a way that database developers can minimize misinterpretation of foods [31]. The EaT app is different from other dietary data collection apps in that it includes a large number of branded ready-to-eat chain items that contribute a large proportion of foods consumed by our target group of young adults. Our database provides access to food names that the public understands, which may improve accuracy beyond that when generic names are used. While nutrition researchers may have less difficulty matching commercial foods to generic foods in a database, this may not be the case for the general public. Newer technology approaches transfer the burden of correctly identifying the food consumed from the researcher to the participant, hence the need for increased “user friendly” food and beverage names in an electronic dietary assessment tool. Similar to previous Australian research on an earlier version of AUSNUT, we renamed most foods and provided branded examples, which increased face validity of the final food names [31].

A database of packaged food and beverages has been developed in the United States [32]. Consistent with the current study, they found some limitations in the US Food Composition database for assessing population trends in the nutrients provided from packaged foods that were continually changing and being updated [32]. In our case, the deficiency is with the inclusion of extensive data on foods prepared outside the home, which appears to be dominant in young adults’ food intake. The US research group was able to demonstrate that the use of the modified database identified changes in the energy density of foods from stores and vending machines that the generic database could not when applied to the same National Health and Nutrition Examination Survey dietary data [32]. This may be important in studying trends in food consumption over time and explanation of changes in population prevalence of overweight and obesity.

The EaT app includes a function to record where participants sourced their foods [13]. The app has been designed specifically with the intention of collecting data on eating food prepared outside the home and is unique in this aspect. Identifying the source of foods may help direct policies. In the state in Australia where the app will be used to collect data, menu labeling legislation is enforced where any chain food outlet with more than 20 stores in the state or 50 in Australia must display energy information for all menu items [33]. However, it is not known if other food outlets exempt from the regulation may make a larger contribution to overall intake of energy and deleterious nutrients. The EaT app will enable the examination of young adults’ eating habits in relation to foods eaten outside the home. This is a research gap here in Australia, and results of the MYMeals Study will be used to shape health promotion messages for improving young adults’ food choices when eating outside the home.

### Strengths and Limitations

As suggested by other researchers, a limitation of food composition databases is that they are correct only at one time-point, and there is an ongoing challenge in updating them [11]. In particular, the ready-to-eat chains constantly offer new, limited-time-only menu items [34], meaning that the app will not include the most recent items. Similarly, the EaT app database is based on the foods consumed as part of the NNPAS, which was collected in 2011-2012 [35]. In 2015, there were 4143 new grocery items introduced in Australian supermarkets [36]. Considering this large number of new items, there will be many items consumed by future participants and these will not be in the database. However, if participants cannot find a food, such as a new ready-to-eat chain or grocery item, the app allows them to manually enter it as a new food. This flags the food to the research team for follow-up. New grocery items will also be identified this way.

Other researchers developing databases to underpin dietary assessment methods can learn from our study. Naming conventions for database entries must contain brand and colloquial names for foods to improve participants’ ability to find foods. Approximate spelling matching functionality or alternative spelling of food names should be incorporated to account for spelling errors. The EaT app is a research tool that could be used to investigate other aspects of young adults’ dietary consumption, for example foods prepared at home, or further tested in other population groups.

In the future, the EaT app will be validated by comparing dietary data collected using the app with 24-hour recalls and used to assess the eating-out habits of 1000 young adults [13]. If finances permit, biomarkers such as doubly labeled water and urinary nitrogens could be used. The EaT app could be further developed for other future purposes, including behavior change programs by incorporating feedback to participants. By validating the app, it can be determined whether an updated database will be required in future versions and uses of the app.

### Conclusions

This paper has described the development of the EaT mobile phone dietary assessment app and the database that underpins it. The database contains comprehensive nutrition information about foods from many large Australian ready-to-eat chains, including commonly eaten portion sizes. Feedback from app usability testing led to updated database naming, enhanced keyword searching, and the addition of functions to enhance usability, such as adding brief instructional screens. There is potential for the features of the EaT app to facilitate the collection of more accurate dietary intake data. The database and the app will be valuable dietary assessment resources for researchers.

## Abbreviations

AUSNUT: AUStralian Food and NUTrient Database
EaT app: Eat and Track app
MYMeals Study: Measuring Young adults’ Meals Study
NNPAS: National Nutrition and Physical Activity Survey
SUS: System Usability Scale
